# Comparative Proteomic Profiling and Biomarker Identification of Traditional Chinese Medicine-Based HIV/AIDS Syndromes

**DOI:** 10.1038/s41598-018-22611-3

**Published:** 2018-03-08

**Authors:** Li Wen, Ye-Fang Liu, Cen Jiang, Shao-Qian Zeng, Yue Su, Wen-Jun Wu, Xi-Yang Liu, Jian Wang, Ying Liu, Chen Su, Bai-Xue Li, Quan-Sheng Feng

**Affiliations:** 1 Chengdu University of Traditional Chinese Medicine, Chengdu, 610075 China; 2 TCM Center for AIDS Prevention and Treatment, China Academy of Chinese Medical Sciences, Beijing, 100700 China; 3 Sichuan Academy of Chinese Medicine Sciences, Chengdu, 610041 China

## Abstract

Given the challenges in exploring lifelong therapy with little side effect for human immunodeficiency virus infection and acquired immune deficiency syndrome (HIV/AIDS) cases, there is increasing interest in developing traditional Chinese medicine (TCM) treatments based on specific TCM syndrome. However, there are few objective and biological evidences for classification and diagnosis of HIV/AIDS TCM syndromes to date. In this study, iTRAQ-2DLC-MS/MS coupled with bioinformatics were firstly employed for comparative proteomic profiling of top popular TCM syndromes of HIV/AIDS: accumulation of heat-toxicity (AHT) and Yang deficiency of spleen and kidney (YDSK). It was found that for the two TCM syndromes, the identified differential expressed proteins (DEPs) as well as their biological function distributions and participation in signaling pathways were significantly different, providing biological evidence for the classification of HIV/AIDS TCM syndromes. Furthermore, the TCM syndrome-specific DEPs were confirmed as biomarkers based on western blot analyses, including FN1, GPX3, KRT10 for AHT and RBP4, ApoE, KNG1 for YDSK. These biomarkers also biologically linked with the specific TCM syndrome closely. Thus the clinical and biological basis for differentiation and diagnosis of HIV/AIDs TCM syndromes were provided for the first time, providing more opportunities for stable exertion and better application of TCM efficacy and superiority in HIV/AIDS treatment.

## Introduction

HIV/AIDS pandemic represents the most severe global health challenge in modern history^1,2^. By the end of 2016, *ca*. 36.7 million people were living with HIV and *ca*. 35.0 million people have died from AIDS-related illnesses worldwide^3^. The development of therapies for prevention and control of HIV/AIDS remains a herculean and significant task.

So far, the highly active antiretroviral therapy (HAART) is the most effective treatment for HIV/AIDS, leading to a notable decrease of mortality rates^4,5^. However, the medication involved in HAART is often limited by drug toxicity, poor treatment tolerability and drug resistance^6^. Traditional Chinese medicine (TCM), with more than 5000 years of clinical practice, has become one of the mainstays of complementary and alternative medication therapy^7^. It has been proved that TCM can reduce the side effects associated with HAART, enhance the immune functions of patients, and improve clinical symptoms and quality of life^8,9^. Furthermore, recent clinical study showed that TCM have positive implications on long-term survival of HIV/AIDS patients^10^, suggesting that TCM has the potential to become a functional cure for HIV/AIDS. Thus TCM is increasingly getting into the clinical application for HIV/AIDS treatments^11,12^.

TCM syndromes, also called ‘*Zheng*’ in Chinese, describe the process of summarizing and distinguishing comprehensive signals and symptoms of individuals from a particular stage of disease^13^. Central to TCM treatment, the differentiation of TCM syndromes is the key principle in guiding the prescription of TCM medications. It aims at specific TCM syndromes and guides the direction of treatment based on TCM theories. Hence accurate discrimination to TCM syndromes is the indispensable prerequisite for effective TCM treatments. However, firstly, the biological evidences for classification of TCM syndromes remain lacking. Moreover, the recognition of TCM syndromes is currently assessed by traditional inspection, auscultation, olfaction, interrogation and palpation^14^. Such method heavily relies on the clinical experiences of TCM practitioners, usually with certain degree of subjectivity and ambiguity from individuals, greatly hindering the stable exertion of TCM efficacy and superiority. TCM treatments of HIV/AIDS also face the problems. Hence it is urgently needed to scientifically prove the differences of TCM syndromes and provide objective diagnosis of HIV/AIDS TCM syndromes. Shao Li *et al*. found the metabolism-immune imbalanced network and potential biomarkers to evaluate cold and hot syndromes^15,16^, which inspired us to explore a new way to biologically understand TCM syndromes. HIV/AIDS TCM syndromes mainly include accumulation of heat-toxicity (AHT) syndrome and Yang deficiency of spleen and kidney (YDSK) syndrome, lung qi and yin deficiency syndrome, qi deficiency and blood stasis syndrome, and original qi and kidney yin deficiency syndrome^17–19^. Among them, AHT and YDSK syndromes account for the top proportions^20,21^ and thus were focused on in our investigation. Patients with AHT syndrome usually present with herpes or aphtha, skin fester or ulcer, sticky shit, and fever *etc*. (Supplementary Table S1), whereas YDSK patients usually accompany symptoms of loose stool, diarrhea, sensation of chill, poor appetite, and edema, *etc*. (Supplementary Table S2).

Comparative serum proteomics has become a robust approach for the demonstration of overall protein levels and identification of disease biomarkers in recent^22–24^. Due to the high sensitivity, accuracy and throughput^25^, the isobaric tags for relative and absolute quantification coupled with two-dimensional liquid chromatography-tandem mass spectrometry (iTRAQ-2DLC-MS/MS) technology has been proposed as a powerful alternative^25–27^. A flurry of applications, *e.g*. detection of diagnosis biomarkers for pulmonary tuberculosis^28^, chronic hepatitis B^29^ and prenatal neural tube defects^30^, have been driven in past decade.

In this research, we aimed to identify the biological differences and serum biomarkers of AHT and YDSK syndromes by employing iTRAQ-2DLC-MS/MS coupled with bioinformatics^23,31^ and western blot analyses^29^. Differentially expressed proteins (DEPs) of AHT and YDSK patients compared with healthy people were screened out with iTRAQ-2DLC-MS/MS firstly. These DEPs were further comparably analyzed with bioinformatics assays *via* Gene Ontology (GO), Kyoto Encyclopedia of Genes and Genomes (KEGG) pathways and protein-protein interaction (PPI) networks. Thus the biological differences between the TCM syndromes can be scientifically dissected and proved. In addition, western blot assays were carried out to measure the level of TCM syndrome-specific DEPs so that the objective diagnosis biomarkers of HIV/AIDS TCM syndromes can be confirmed.

## Results

### Comparative serum proteomic profiling of AHT and YDSK syndromes by iTRAQ-2DLC-MS/MS

**Figure 1:**
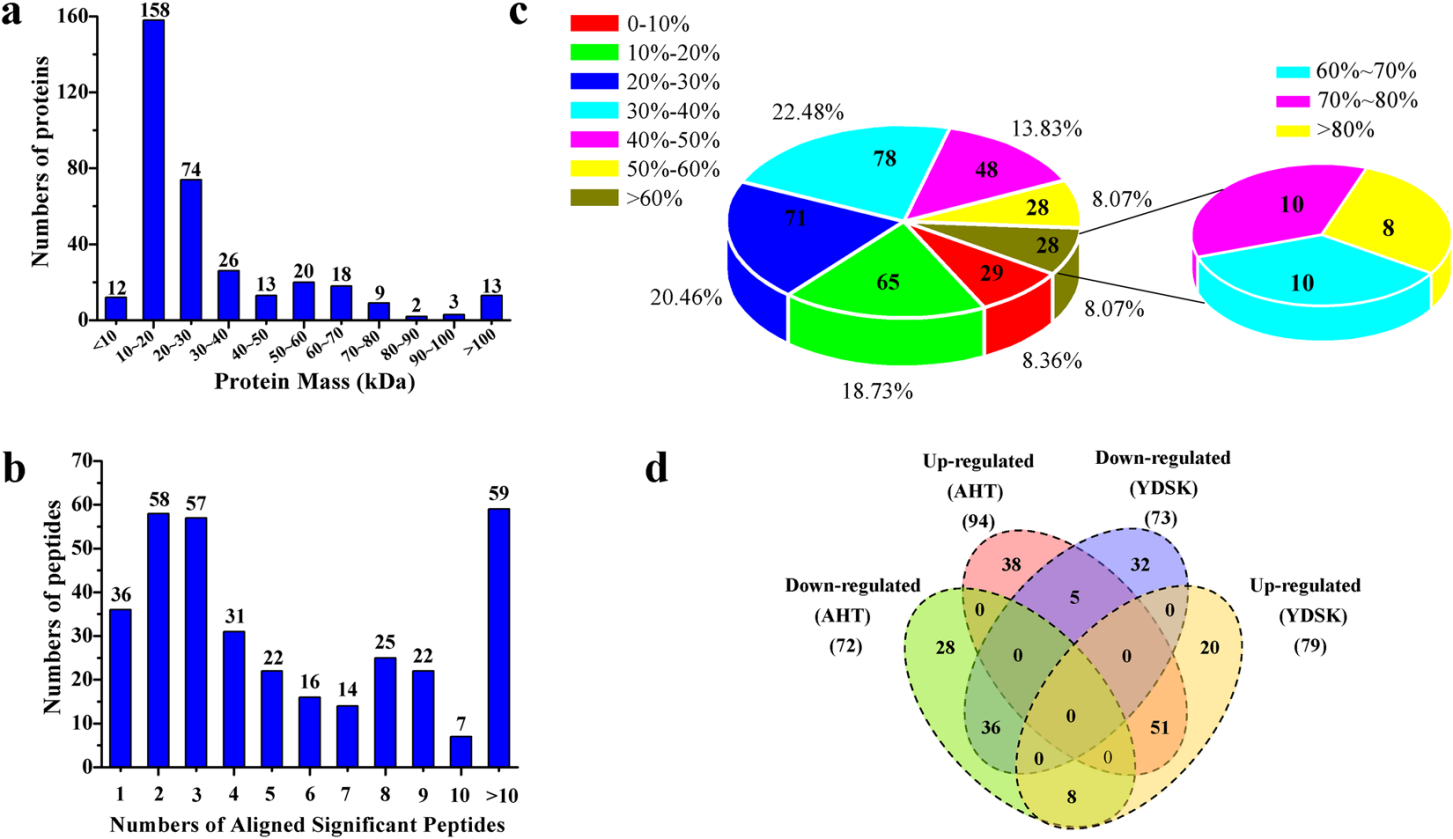
Identification and analysis of serum proteome of HIV/AIDS patients. (**a**) Distribution of protein mass of identified proteins. (**b**) Number of proteins aligned with significant peptides. (**c**) Coverage of the identified proteins. (**d**) Venn diagram of up-regulated and down-regulated proteins in AHT and YDSK groups.

To gain biological insights into the differences of TCM syndromes and further discover serum protein biomarkers, 45 participants were obtained for each TCM syndrome, including 15 AHT patients, 15 YDSK patients and 15 healthy volunteers as the control. The clinical baseline data (Supplementary Table S3) showed that the participants were available for the next comparative serum proteomic profiling with iTRAQ-2DLC-MS/MS. The mass distribution of the identified proteins (Fig. 1a) revealed that 336 (96.54%) were above 10 kDa, of which 158 (45.53%) were 10 to 20 kDa and 13 (3.74%) were above 100 kDa. According to the statistics of the proteins aligned with significant peptides (Fig. 1b), 311 (89.62%) proteins were aligned by more than two peptides. In addition, the coverage of protein sequence with 50~100%, 40~50%, 30~40%, 20~30%, 10~20%, and under 10% accounted for 24.50%, 13.83%, 22.48%, 20.46%, 18.73% and 8.36%, respectively (Fig. 1c).

Furthermore, according to the quantitative analysis of the identified proteins, those showed greater than 1.2-fold change (up-regulated) or less than 0.8-fold change (down-regulated) were screened out as the DEPs (Supplementary Table S4 and Table S5). Venn diagram^32^ (Fig. 1d) revealed that 166 DEPs were identified in AHT, including 94 up-regulated and 72 down-regulated proteins. 152 DEPs including 79 up-regulated and 73 down-regulated proteins were obtained in YDSK. Among these DEPs, AHT and RDSK syndromes shared 51 up-regulated and 36 down-regulated proteins, respectively. In addition, 5 proteins were identified with up-regulation in AHT but with down-regulation in YDSK, while the expressions of 8 proteins were up-regulated in YDSK but were down-regulated in AHT (Supplementary Table S6). Moreover, there were 56 and 52 DEPs specifically involved in AHT and YDSK, respectively.

### Comparative GO analyses of the DEPs in AHT and YDSK syndromes

**Figure 2:**
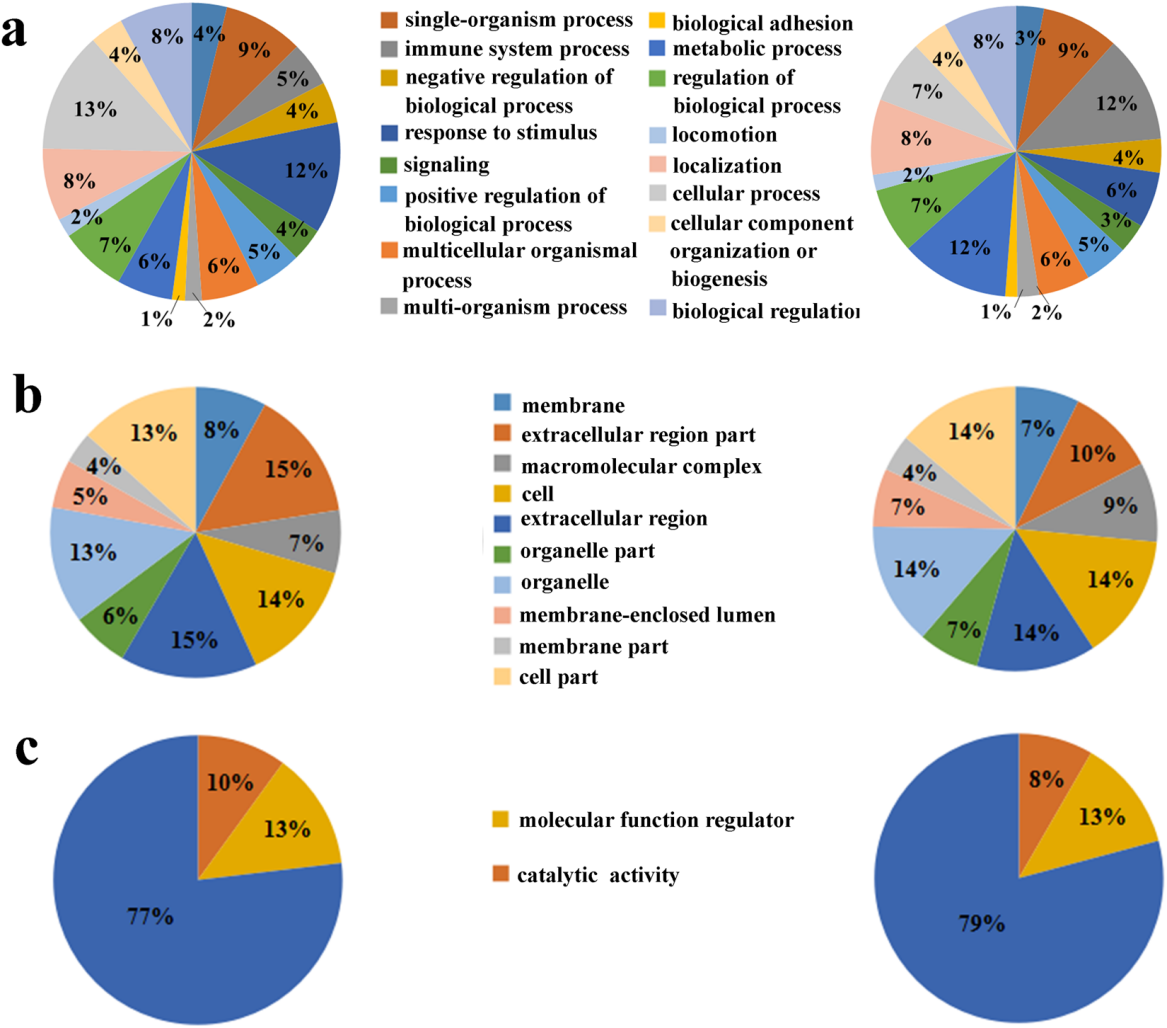
GO annotation of DEPs of AHT (left) and YDSK (right). (**a**) Biological process. (**b**) Cellular component. (**c**) Molecular function.

To deeply dissect the biological differences between AHT and YDSK syndromes, intensive bioinformatics analyses were carried out. In consideration of that the classification and specificity of TCM syndromes are related to the biological regulation of all the DEPs, 166 DEPs in AHT and 152 DEPs in YDSK were used for bioinformatics analyses respectively. Broad functional distribution according to GO analyses^31^ showed that the DEPs in AHT and YDSK shared the same categories in biological process (Fig. 2a), cellular component (Fig. 2b) and molecular function (Fig. 2c). However, the proportional distributions of the categories for the two TCM syndromes were different. Biological process analyses revealed that the DEPs in AHT were mainly localized to response to stimulus (12%) and cellular process (13%), while metabolic process (12%) and immune system process (12%) accounted for a large proportion in YDSK (Fig. 2a). In cellular component analyses, DEPs in AHT were mainly localized to extracellular region part (15%), extracellular region part (15%) and cell (14%), whereas there were much fewer DEPs located to extracellular region part (10%) in YDSK (Fig. 2b).

### Comparative KEGG pathway analyses of the DEPs in AHT and YDSK syndromes

**Figure 3:**
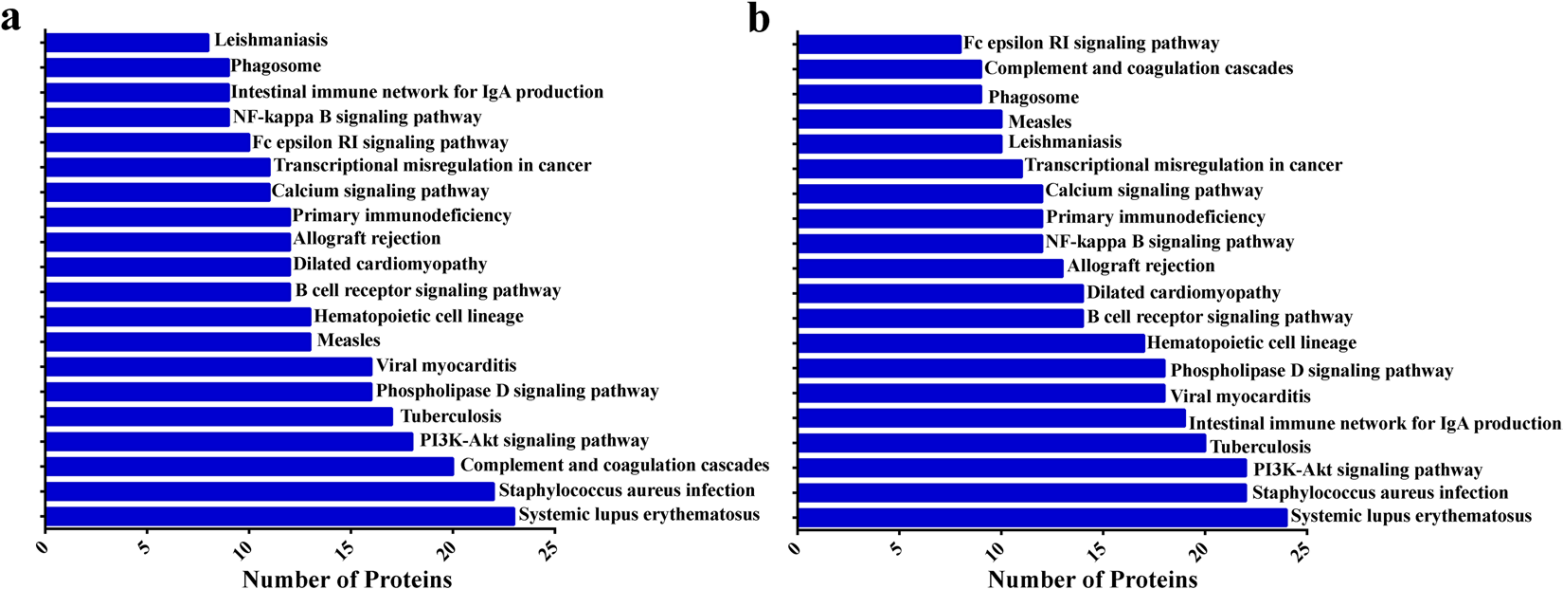
KEGG pathway analyses of the DEPs in AHT (**a**) and YDSK (**b**) groups.

KEGG pathway annotation^29^ revealed that the DEPs in AHT and YDSK group both mainly functioned in the signaling pathways of systemic lupus erythematosus (hsa05322), staphylococcus aureus infection (hsa05150), tuberculosis (hsa05152), viral myocarditis (hsa05416), PI3K-Akt signaling pathway (hsa04151) and phospholipase D signaling pathway (hsa04072) (Fig. 3). Notably, DEPs linked to the pathway of complement and coagulation cascades (hsa04610) in AHT were much more than those in YDSK, whereas more DEPs were associated with the pathway of intestinal immune network for IgA production in YDSK compared to those in AHT.

### Comparative STRING analyses of the DEPs in AHT and YDSK syndromes

**Figure 4:**
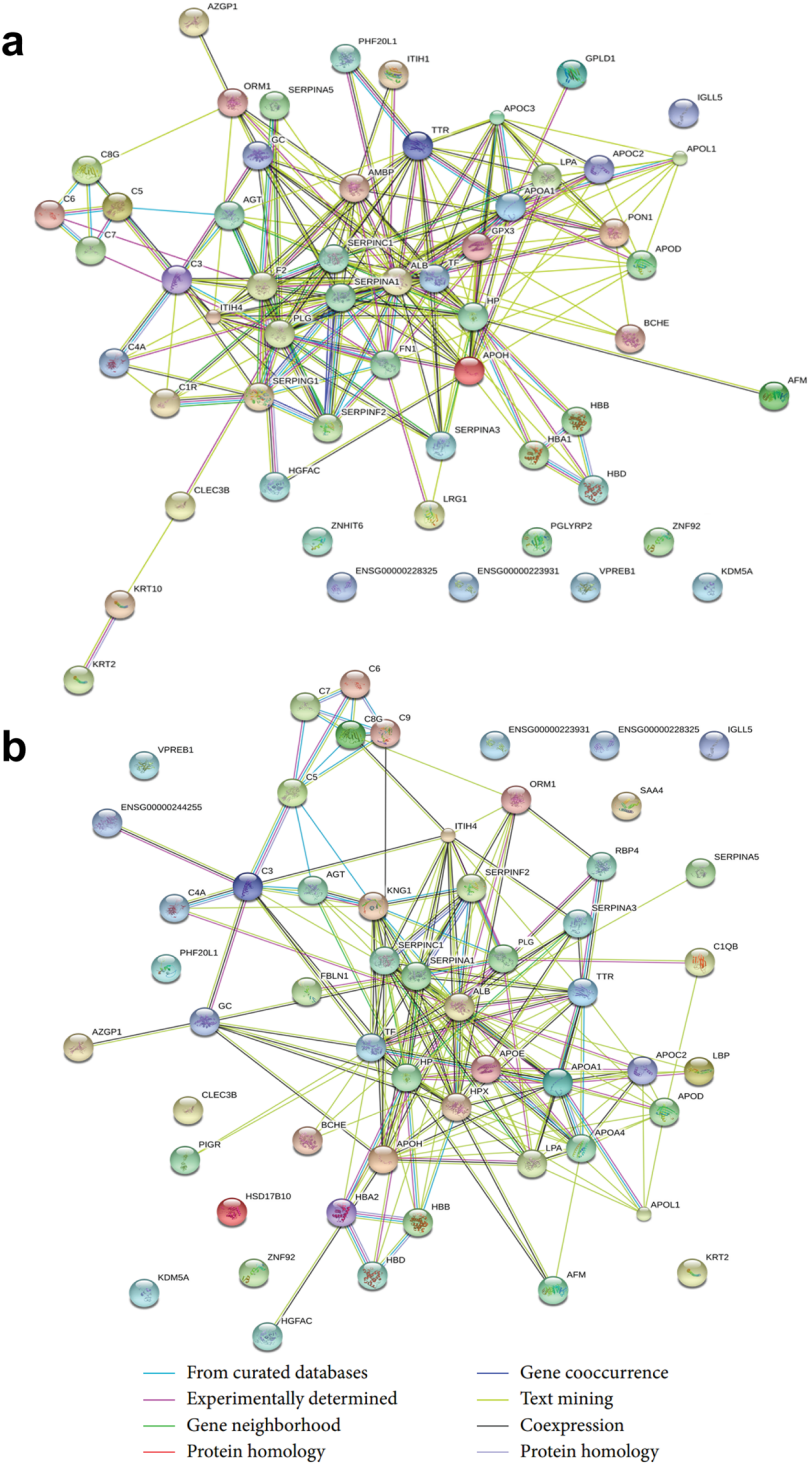
PPI network of the DEPs in AHT (**a**) and YDSK (**b**).

Furthermore, STRING analyses of PPI networks^31,33^ displayed the significant DEPs involved in physical and functional connections (Fig. 4). Among these DEPs, quite a number of proteins specifically functioned in response to AHT and YDSK syndromes respectively. For example, fibronectin 1 (FN1), glutathione peroxidase 3 (GPX3), apolipoprotein C3 (APOC3), alpha-1-microglobulin/bikunin precursor (AMBP) and keratin 10 (KRT10) were specifically involved in the PPI network in AHT group (Fig. 4a), while apolipoprotein E (ApoE), retinol binding protein 4 (RBP4), lipid-binding protein (LBP), Kininogen 1 (KNG1), Fibulin 1 (FBLN1) and alpha-2-glycoprotein 1, zinc-binding (AZGP1) were specifically associated with the PPI network in YDSK group (Fig. 4b). These DEPs might be potential biomarkers for identifying and distinguishing AHT and YDSK syndromes of HIV/AIDS.

### Validation of the biomarkers for AHT and YDSK syndromes

**Figure 5:**
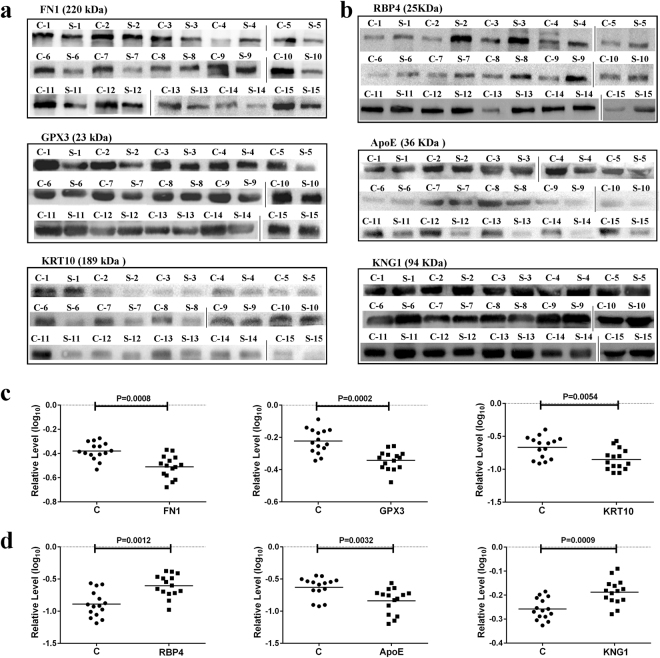
Validation of the biomarkers for AHT and YDSK syndromes. (**a**,**b**) Western blot analyses of FN1, GPX3 and KRT10 in AHT (a), and RBP4, ApoE and KNG1 in RSYX (b). The grouping of bands cropped from different gels was made explicit using delineation with dividing lines. (**c**,**d**) Statistical analyses of the relative levels of FN1, GPX3 and KRT10 (c) corresponding to (a), and RBP4, ApoE and KNG1 (d) corresponding to (d) (n = 15). Median values are shown by a horizontal line. C = control.

To validate iTRAQ-2DLC-MS/MS results and further identify the biomarkers of TCM syndromes, TCM syndrome-specific DEPs in PPI network of each syndrome were selected for western blot assays. The protein bands (Fig. 5a) and the corresponding statistical analyses (Fig. 5c) showed that the serum levels of FN1, GPX3 and KRT10 in AHT group were significantly lower than that of the healthy control, with the fold changes of 0.71, 0.63, 0.74 and *p* value of 0.0008, 0.0002, 0.0054, respectively. For YDSK group, the serum levels of RBP4 and KNG1 were much higher but ApoE was significantly lower than those of the control, with the fold changes of 1.47, 0.72, 1.91 and *p* = 0.0008, *p* = 0.0009, *p* = 0.0032, respectively (Fig. 5b and d). These results were consistent with iTRAQ-2DLC-MS/MS data. More importantly, it was further confirmed that FN1, GPX3 and KRT10 can be employed as AHT biomarkers, and that RBP4, ApoE and KNG1 were available YDSK biomarkers.

## Discussion

Although TCM has been long practiced effectively for HIV/AIDS treatment, objective and biological evidences for classification and diagnosis of the TCM syndromes are still lacking to date. As the top popular TCM syndromes of HIV/AIDS, AHT and YDSK were investigated in this study. According to serum proteomic analyses with iTRAQ-2DLC-MS/MS, the identified DEPs in the two TCM syndromes were significantly different, with 56 and 52 DEPs specifically linked to AHT and YDSK respectively. The proteomic profiles indicated that there were biological differences between the AHT and YDSK syndromes, which were further confirmed and dissected by bioinformatics analyses including GO, KEGG and PPI network.

For the two TCM syndromes, GO data revealed that the proportional distributions of their DEPs in biological process and cellular component were inconsistent. In comparative KEGG pathway analyses, complement and coagulation cascades pathway linked more closely with AHT than with YDSK. In TCM, AHT syndrome of HIV/AIDS was regarded as the stage when virus and immune system confront each other, and therefore inflammatory reaction signs such as fever and aphtha are the major symptoms of AHT patients. Comparatively, YDSK syndrome is the stage when anti-virus immune reaction became weaker, and thus it is associated with the symptoms of chill and loose stool, *etc*. It has been reported that when the complement system was activated, the effect fragments of inflammatory medium can be generated and subsequently involved in inflammatory response. Thus the complement system was the first line of defense against virus invasion^34^. These are consistent with the close connection of complement and coagulation cascades pathway with AHT syndrome in the KEGG analysis. Moreover, previous studies have revealed that IgA is protective for the integrity of the intestinal barrier^35^, which has an important effect on antigen elimination. KEGG analyses in our study showed that the pathway of intestinal immune network for IgA production was more related with YDSK than with AHT, indicating there were differences of intestinal immune function between the two TCM syndromes. This result is accordant with the typical clinical symptoms of YDSK such as abdominal distention and loose stool. All these above further validated the iTRAQ-2DLC-MS/MS data and the biological differences between AHT and YDSK syndromes.

Identification of TCM syndrome biomarkers is of great importance for objective diagnosis of HIV/AIDS TCM syndromes and the subsequent accurate TCM treatment. In this work, based on the credibility of the iTRAQ-2DLC-MS/MS results, the TCM syndrome-specific DEPs in the PPI networks were further validated as the biomarkers, including FN1, GPX3, KRT10 for AHT and RBP4, ApoE, KNG1 for YDSK, respectively.

FN1 can help macrophage grab and clear the damaged cells and harmful substances, and thus has important regulating effect on systemic inflammatory response^36,37^. AHT patients usually present inflammatory response related symptoms and physical signs, which was in accordance with the reduction of FN1 in our experiments. GPX3 is a critical antioxidant enzyme for reducing reactive oxygen species and maintaining the oxygen balance^38^. The infection and replication of HIV viruses can lead to oxidative damage^39^ and the subsequent reduction of GPX3^40,41^. In our experiments, GPX3 was specifically down-regulated in AHT syndrome. This might be caused by the severe oxidative damage in AHT syndrome, since the infection and replication of HIV in AHT syndrome is more intensive than that in YDSK syndrome and healthy individuals. KRT10 can be released from the epithelial cell sourced tumor cell during the proliferation and apoptosis, and thus has been used as biomarker for clinical diagnosis of epithelial cell tumor^42^. In TCM practice on HIV/AIDS, it was found that the incidence of epithelial cell tumor in AHT syndrome was much lower compared with YDSK syndrome, which was consistent with the low expression in AHT case in our experiments. As the most important extracellular transport protein^43^, RBP4 is closely related to metabolic dysfunction^44–46^. RBP4 levels are higher in patients with clinical hypothyroidism and exhibit an obvious decrease after normalization of thyroid function^47^. YDSK patients performed certain hypothyroidism symptoms such as sensation of chill, debilitation, edema, poor appetite, sexual dysfunction, which might closely linked to the increased RBP4 level in our experiments. ApoE has been reported to be a risk factor for vascular dementia and Alzheimer’s disease. Researchers have found that ApoE was closed related to HIV-associated neurocognitive disorder^48^. HIV/AIDS YDSK patients usually exhibited apathetic expression and decreased memory, which may be caused by the abnormal expression of ApoE. Previous studies^49,50^ have showed that KNG1 can enter into the infected tissues, interact with cell matrix and be subsequently degraded to kinin, which can activate the dendritic cell. Then Th1-type and adaptive responses can be induced. Therefore, KNG1 level in tissues is positively related with the immune response ability. KNG1 in blood plasmas of YDSK patients was increased in our experiments, indicating the down-regulation of KNG1 in tissues and the reduction of immune response ability. This was in accordance with the YDSK symptoms similar to immune tolerance state, such as sensation of chill and diarrhea. Taken together, these validated DEPs were biologically and specifically related to the TCM syndromes and thus can serve as potential AHT or YDSK biomarkers of HIV/AIDS.

However, there also exist some limitations in the study. The number of the enrolled patients with each syndrome is insufficient. Some powerful bioinformatics analyses such as Ingenuity Pathway Analysis^51^ and MetaCore Analysis^52^ were not performed. Gene expression^33^ and metabolic profiling^53^ of HIV/AIDS should be further carried out. The molecular regulating mechanisms of the identified DEPs in each syndrome need to be dissected in further studies. Other TCM syndromes should also be investigated in future to comprehensively understand TCM syndromes of HIV/AIDS.

## Conclusion

Based on iTRAQ-2DLC-MS/MS discovered DEPs and the further bioinformatics analyses, it was found that there were biological differences between the top popular TCM syndromes of HIV/AIDs: AHT and YDSK. The differences included the kinds of identified DEPs and their function distributions in cellular components and biological processes, as well as the pathways that the DEPs participated in. The dissections of these differences provide clinical and experimental evidences for the differentiation of TCM syndromes for the first time. Furthermore, TCM syndrome-specific DEPs were identified as the serum biomarkers, including FN1, GPX3, KRT10 for AHT and RBP4, ApoE, KNG1 for YDSK. These biomarkers also biologically linked with the specific TCM syndromes closely and thus can be used for TCM syndrome determination. All these laid biological basis for the differentiation and diagnosis of HIV/AIDs TCM syndromes with important scientific significance, providing more opportunities for deep understanding of TCM syndromes and stable exertion of TCM superiority in HIV/AIDS treatment.

## Methods

The present study was approved by the research medical ethics committee of Chengdu University of TCM (China), and signed informed consent was obtained from all participants. All methods were performed in accordance with the relevant guidelines and regulations.

### Clinical evaluation

Both western and Chinese medicine criteria were used to divide HIV/AIDS patients into two groups. First, participants were satisfied with diagnostic criteria of western medicine derived from ‘*AIDS Treatment Guidelines (2011 version)*’ Then, AHT and YDSK patients were diagnosed according to ‘*Traditional Chinese Medicine Treatment of AIDS Clinical Technology Program (2004 Edition)*’. Patients were filtered when they satisfied one of the following criteria: pregnant women or nursing mothers; with malignant tumors; with unconsciousness, dementia or mental disease; family members disagree; with primary immunodeficiency or other secondary immunodeficiency caused by hormone chemotherapy; with hematological disorders; with central nervous system diseases or severe organ diseases that were not caused by HIV/AIDS.

### Patients and serum collection

A total of 87 HIV/AIDS patients (aged 21 to 61 years) were obtained from Chengdu infectious disease hospital, where is the demonstration area for prevention and control of national infectious disease in Liangshan prefecture. These patients were filtered with clinical evaluation as described above. In addition, 35 randomly chosen healthy volunteers, aged from 24 to 54 years were also included (Supplementary Table S3). For both iTRAQ-2DLC-MS/MS proteomics and western blot analyses, blood samples of 15 AHT-HIV/AIDS patients, 15 YDSK-HIV/AIDS patients and 15 healthy participants were obtained. Serum was collected from blood sample (4 mL) following the manufacture’s protocol. Briefly, each blood sample was incubated at room temperature for 2 h in vacutainer blood handling tube (Becton Dickinson, New Jersey, USA) and centrifuged for 10 min at 3500 rpm and 4 °C. The supernatants were collected as serum sample, transferred into polypropylene tube and stored at −80 °C.

### Protein preparation

200 *μ*L of serum sample from each patient was subjected to reduce the complexity by using ProteoMiner^TM^ Kits (Bio-Rad Laboratories, Hercules, CA, USA) according to the manufacturer’s instructions. The prepared proteins were stored at −80 °C for subsequent analysis. Protein concentration was determined using the Bradford protein assay (Bio-Rad Protein Assay; Bio-Rad Laboratories, Hercules, CA, USA).

### iTRAQ-2DLC-MS/MS discovery experiments

Proteins from 15 patients in the same group were pooled for iTRAQ labelling. Pooled protein samples (100 *μ*g) were digested with Trypsin Gold (Promega, Madison, WI, USA) with the ratio of protein:trypsin = 30:1 at 37 °C for 16 h. Digested peptides were dried by vacuum centrifugation, reconstituted in 0.5 M TEAB and processed in preparation for 8-plex iTRAQ (AB Sciex, Framingham, MA, USA). The peptides were labeled with isobaric tags and incubated for 2 h at room temperature. The labeled peptide mixtures were then pooled, dried by vacuum centrifugation and separated by strong cation exchange (SCX) chromatography on a 20AD high-performance liquid chromatography (HPLC) system (Shimadzu, Kyoto, Japan).

The fractions were resuspended in buffer A (water, 0.1% FA) and centrifuged at 20000 g for 10 min. The supernatant was loaded onto a C18 trap column (2 cm) on a LC-20AD nanoHPLC (Shimadzu, Kyoto, Japan) and eluted onto an analytical C18 column (10 cm, inner diameter 75 *μ*m). The samples were loaded with buffer B (ACN, 0.1% FA) at 400 nL/min. MS data acquisition was performed with a Q-Exactive (Termo Fisher Scientifc, San Jose, CA).

### Protein identification and quantification

The raw data files were processed and quantified with Proteome Discoverer software version 1.3 (Thermo). The MASCOT version 2.3.0 search engine was used for all searches. All searches were performed against the human protein sequence database (NCBInr_2014_human). For protein identification, the following criteria were used: trypsin digestion allowing a maximum of one missed cleavages; Carbamidomethyl (C) was set as fixed modifications. Oxidation (M), Gln → Pyro-Glu (N-term Q, iTRAQ 8 plex (K), iTRAQ 8 plex (Y), iTRAQ 8 plex (N-term) were selected as variable modifications. The peptide mass tolerance and MS/MS tolerance were set to ±15 ppm and 20 mmu, respectively.

The quantification was processed using Proteome Discoverer version 1.3 with the following settings: the fold change threshold for up-/down-regulation was set at 20; only peptides unique for a given protein or protein group and with the 99% confidence were selected for quantitation; the number of the minimum unique peptides was set at one; experimental bias was corrected by normalization to the median in each sample. Venn diagram of up- and down-regulated proteins was analyzed by InteractiVenn (http://www.interactivenn.net/).

### Bioinformatics analyses

GO annotation proteome was derived from the UniProt-GOA Database (www. http://www.ebi.ac.uk/GOA/). Proteins were classified by GO annotation based on three categories: biological process, cellular compartment and molecular function. KEGG database were used to annotate pathways. First, we annotate proteins using KEGG online service tools KAAS. Then we map the annotation result on the KEGG database using KEGG online service tools KEGG mapper. PPI were analyzed for identified acetylated proteins using Cytoscape software. PPI network obtained from STRING database, a weighted interaction database with physical and functional interactions^33^. We fetched all interactions that had a confidence score ≥0.7 (high confidence).

### Western blot and statistical analyses

The prepared protein samples (10 *μ*L) from 15 AHT-HIV/AIDS patients, 15 YDSK-HIV/AIDS patients and 15 healthy individuals were treated with gel-loading buffer for SDS-PAGE electrophoresis at 120 V for 1 h. The samples were then transferred to a PVDF membrane (Amersham Biosciences) at 15 V for 20 min. The membranes were blocked with 5% bovine serum albumin at 37 °C for 2 h and then incubated with primary antibodies (including anti-FN1, anti-GPX3, anti-KRT10, anti-RBP4, anti-ApoE and anti-KNG1, 1:500, Santa Cruz Biotechnology, CA, USA) at 4 °C overnight, followed by incubation with alkaline phosphatase-conjugated secondary antibodies (1:1000) at 37 °C for 1 h in darkness. The bands were visualized with enhanced chemiluminescence (GE Healthcare) and the band intensities were quantified with ImageJ (Wayne Rasband, National Institutes of Health). Albumin was used as reference to calculate the relative intensity of each protein. The calculation of mean ± standard deviation and Students’ *t* tests were performed with GraphPad Prism 6.0 software.

## Electronic supplementary material

Supplementary Information
