# Supplementary material for: Comparative Proteomic Profiling and Biomarker Identification of Traditional Chinese Medicine-Based HIV/AIDS Syndromes
Source: Sci Rep. 2018 Mar 8;8:4187. doi: 10.1038/s41598-018-22611-3 (PMC5843661; doi:10.1038/s41598-018-22611-3)
Supplement: Supplementary file 1 — Supplementary Information [file 41598_2018_22611_MOESM1_ESM.docx]

**Supplementary Information**

**Comparative** **Proteomic Profiling and Biomarker Identification of Traditional Chinese Medicine-Based HIV/AIDS Syndromes**

Li Wen^1,^*, Ye-Fang Liu^1,^*, Cen Jiang^1^, Shao-Qian Zeng^1^, Yue Su^1^, Wen-Jun Wu^1^, Xi-Yang Liu^1^, Jian Wang^2^, Ying Liu^2^, Chen Su^3^, Bai-Xue Li^1^, and Quan-Sheng Feng^1^

^1^Chengdu University of Traditional Chinese Medicine, Chengdu 610075, China

^2^TCM Center for AIDS Prevention and Treatment, China Academy of Chinese Medical Sciences, Beijing 100700, China

^3^Sichuan Academy of Chinese Medicine Sciences, Chengdu 610041, China

*These authors contributed equally to this work

Correspondence and requests for materials should be addressed to Q.S.F. (email: [fengqs118@163.com](mailto:fengqs118@163.com)) or B.X.L (email: [baixuelee@163.com](mailto:baixuelee@163.com))

1. **Diagnosis scale for AHT and YDSK syndromes of HIV/AIDS**

The Diagnosis scale for AHT and YDSK syndromes of HIV/AIDS referred to *Clinical Technical Plan for Chinese Medicine Treatment of AIDS (**Trails)* *(Edition 2004)*.

Table S1. Diagnosis scale for AHT syndrome of HIV/AIDS

| Symptom | Symptom classifications | | | | Weighing Coefficient | Symptom Integral |
| --- | --- | --- | --- | --- | --- | --- |
|  | No (0) | Mild (1) | Medium (2) | Severe (3) |  |  |
| Refractory herpes or aphtha | □ | □ | □ | □ | 5 |  |
| Skin fester or ulcer | □ | □ | □ | □ | 4 |  |
| Heaviness in head and body | □ | □ | □ | □ | 3 |  |
| Sticky shit | □ | □ | □ | □ | 3 |  |
| Bitterness in mouth | □ | □ | □ | □ | 3 |  |
| Mouth dryness | □ | □ | □ | □ | 2 |  |
| Distention and fullness | □ | □ | □ | □ | 2 |  |
| Unsurfaced fever | □ | □ | □ | □ | 1 |  |
| Yellow urine | □ | □ | □ | □ | 1 |  |
| Red tongue |  | yes□ | no□ |  | 2 |  |
| Yellow or greasy coated tongue |  | yes□ | no□ |  | 2 |  |
| Yellow-and-white coated tongue |  | yes□ | no□ |  | 2 |  |
| Number of slippery pulse |  | yes□ | no□ |  | 1 |  |
| Total integral | | | | | | |
| Patients with total integral of above 12 are diagnosed with AHT syndrome | | | | | | |

Table S2. Diagnosis scale for YDSK syndrome of HIV/AIDS

| Symptom | Symptom classifications | | | | Weighing Coefficient | Symptom Integral |
| --- | --- | --- | --- | --- | --- | --- |
|  | No (0) | Mild (1) | Medium (2) | Severe (3) |  |  |
| Loose stool | □ | □ | □ | □ | 5 |  |
| Diarrhoea | □ | □ | □ | □ | 4 |  |
| Sensation of chill, cold limbs | □ | □ | □ | □ | 4 |  |
| Crymodynia in waist and abdomen | □ | □ | □ | □ | 3 |  |
| Edema | □ | □ | □ | □ | 3 |  |
| Poor appetite and anorexia | □ | □ | □ | □ | 2 |  |
| Aching lumbus and limp knees | □ | □ | □ | □ | 2 |  |
| Hearing loss or tinnitus | □ | □ | □ | □ | 1 |  |
| Inhibited urination | □ | □ | □ | □ | 1 |  |
| Pallid complexion | □ | □ | □ | □ | 1 |  |
| Debilitation and disinclination to talk |  | yes□ | no□ |  | 1 |  |
| Pale and fat tongue |  | yes□ | no□ |  | 2 |  |
| White coated tongue |  | yes□ | no□ |  | 2 |  |
| Total integral | | | | | | |
| Patients with total integral of above 12 are diagnosed with YDSK syndrome | | | | | | |

**2. Clinical diagnosis of patients who participated in this study**

ForAHT and YDSK patients, the gender, age andbiochemical indexes except alanine transaminase and viral load were not significantly different (Supplementary Table S1). Mean ages of AHT-HIV/AIDS, PSYX-HIV/AIDS and healthy controls were 40.13, 37.21 and 38.70 years, respectively. Viral load levels in the blood samples of AHT and YDSK patients were ranged from 20 to 2.01E+06cp/mL and 6 to 2.18E+05 cp/mL, respectively. CD4 levels in the blood samples of HIV/AIDS patients were ranged from105 to 540/*μ*L and 101 to 602/*μ*L. In addition,we examined blood lipids, liver and kidney function. The important indicators in these inspections are within the normal range, such as WBC, RBC, HB, ALT, AST and STB, *etc*. In addition, there was no hepatic and renal dysfunction in both AHT and YDSK patients.

Table S3. Clinical diagnosis of patients who participated in this study

|  | Unit | AHT (n=15) | YDSK (n=15) | Control (n=15) |
| --- | --- | --- | --- | --- |
| Sex (female/male) |  | 6/9 | 9/6 | 7/8 |
| Age range  (Mean ± SD) | years | 21～61  (40.13±13.86） | 20～56  (37.21±8.54) | 24～54  (38.70 ± 11.48) |
| White blood cell (Mean ± SD) | 10^9 /L | 3.10～10.6  (5.73 ± 2.17) | 3.7～6.9  (5.043 ± 1.056) |  |
| Red blood cell  (Mean ± SD) | 10^12 /L | 4.19～6.50  (5.08 ± 0.69） | 3.93～5.45  (4.68 ± 0.45) |  |
| Hemoglobin  (Mean ± SD) | g/L | 128～178  (142.0 0 ± 11.93) | 112～170  (150.21 ± 12.45) |  |
| Blood platelet  (Mean ± SD) | 10^9 /L | 29～305  (164.07 ± 74.29) | 4.57～342  (156.47 ± 76.02) |  |
| Leukomonocyte (Mean ± SD) | 10^9 /L | 0.99～55.30  (30.64 ± 14.79) | 1.58～38.6  (26.37 ± 11.52) |  |
| Alanine transaminase (Mean ± SD) | IU/L | 3～46  (11.34 ± 4.31) | 6～67  (39.13 ± 14.39) |  |
| Aspartate transaminase  (Mean ± SD) | IU/L | 14～42  (23.20 ± 8.55) | 16～50  (26.13 ± 8.87) |  |
| Albumin  (Mean ± SD) | g/L | 34～48  (46.46 ± 4.94) | 38～49  (45.38 ± 3.69) |  |
| Total bilirubin (Mean ± SD) | *μ*mol/L | 2.1～20.2  (11.78 ± 4.99) | 5.8～16.2  (12.34± 3.77) |  |
| Blood urea nitrogen (Mean ± SD) | mmol/L | 3.76～8.78  (6.40 ± 1.14) | 4.48～7.86  (6.25 ± 0.87) |  |
| Triglyceride (Mean ± SD) | mmol/L | 0.90～2.60  (1.57 ± 0.59) | 0.90～4.35  (1.78 ± 0.92) |  |
| Viral load (Mean ± SD) | cp/mL | 20～2014000  (187904.23 ± 754509.46) | 6～218000  (23536.97 ± 56741.59) |  |
| CD4 (Mean ± SD) | /*μ*L | 101～602  (335.80 ± 154.33) | 105～540  (251.07 ± 122.84) |  |
| CD8 (Mean ± SD) | /*μ*L | 270～3111  (1041.8 ± 568.28) | 249～1397 (959.87 ± 327.14) |  |

**3. The differentially expressed proteins (DEPs) in AHT and YDSK patiensts**

Table S4.The DEPs in AHTpatients (part)

| Accession | Description | Score | | Coverage | | # Proteins | | # Peptides | | fold change | |  |
| --- | --- | --- | --- | --- | --- | --- | --- | --- | --- | --- | --- | --- |
| 5524087 | lambda 1 immunoglobin light chain variable region [Homo sapiens] | | 60.56 | | 32.54% | | 295 | | 3 | | 13.586 | |
| 21669541 | immunoglobulin lambda light chain VLJ region [Homo sapiens] | | 415.73 | | 43.91% | | 435 | | 10 | | 4.524 | |
| 21669601 | immunoglobulin lambda light chain VLJ region [Homo sapiens] | | 327.6 | | 36.00% | | 314 | | 8 | | 4.113 | |
| 3142659 | immunoglobulin lambda light chain variable region [Homo sapiens] | | 68.9 | | 36.45% | | 75 | | 2 | | 3.581 | |
| 185364 | immunoglobulin lambda-chain [Homo sapiens] | | 433.52 | | 48.94% | | 509 | | 11 | | 3.409 | |
| 21669319 | immunoglobulin kappa light chain VLJ region [Homo sapiens] | | 473.44 | | 35.88% | | 240 | | 9 | | 2.627 | |
| 58222850 | anti-tetanus toxoid immunoglobulin light chain variable region [Homo sapiens] | | 117.13 | | 37.96% | | 448 | | 3 | | 2.616 | |
| 524454182 | IgG L chain [Homo sapiens] | | 333.42 | | 38.89% | | 7 | | 8 | | 2.613 | |
| 21669331 | immunoglobulin kappa light chain VLJ region [Homo sapiens] | | 379.06 | | 40.37% | | 449 | | 8 | | 1.912 | |
| 42543650 | Chain L, Crystal Structure Of Human Anti-hiv-1 Gp120-reactive Antibody E51 | | 408.68 | | 54.46% | | 110 | | 10 | | 1.893 | |
| 18025654 | immunoglobulin light chain variable region [Homo sapiens] | | 55.66 | | 32.74% | | 511 | | 2 | | 1.885 | |
| 358439859 | Chain L, Crystal Structure Of Hiv-1 Neutralizing Antibody Ch04 | | 470.77 | | 48.37% | | 173 | | 8 | | 1.84 | |
| 18378785 | immunglobulin light chain variable region [Homo sapiens] | | 26.97 | | 15.45% | | 1 | | 1 | | 1.832 | |
| 444737707 | immunoglobulin light chain lambda, partial [Homo sapiens] | | 36.36 | | 13.38% | | 1 | | 1 | | 1.771 | |
| 109693188 | immunoglobulin light chain variable region [Homo sapiens] | | 77.78 | | 36.45% | | 24 | | 2 | | 1.5 | |
| 323433260 | immunoglobulin variable region [Homo sapiens] | | 235.64 | | 36.30% | | 114 | | 5 | | 1.463 | |
| 323432682 | immunoglobulin variable region [Homo sapiens] | | 165.07 | | 26.03% | | 438 | | 4 | | 1.444 | |
| 145942169 | immunoglobulin heavy chain variable region [Homo sapiens] | | 90.26 | | 35.71% | | 2998 | | 3 | | 1.424 | |
| 9857759 | recombinant IgG4 heavy chain [Homo sapiens] | | 467.12 | | 34.96% | | 57 | | 15 | | 1.325 | |
| 170684332 | immunoglobulin lambda 1 light chain [Homo sapiens] | | 88.28 | | 19.53% | | 76 | | 2 | | 1.324 | |
| 18025588 | immunoglobulin light chain variable region [Homo sapiens] | | 163.64 | | 37.96% | | 451 | | 3 | | 1.312 | |
| 86438980 | immunoglobulin heavy chain [Homo sapiens] | | 167.35 | | 22.37% | | 32 | | 4 | | 1.311 | |
| 114147764 | immunoglobulin heavy chain variable region [Homo sapiens] | | 113.32 | | 27.69% | | 283 | | 3 | | 1.286 | |
| 484973 | Ig kappa chain V region (clone LUNm03) - human (fragment) | | 48.47 | | 24.32% | | 2 | | 2 | | 1.282 | |
| 110626504 | anti-SARS-CoV S protein immunoglobulin kappa light chain [Homo sapiens] | | 443.73 | | 43.93% | | 169 | | 8 | | 1.279 | |
| 293651977 | Chain L, Structure Of Human Anti Hiv 21c Fab | | 455.05 | | 59.91% | | 507 | | 11 | | 1.277 | |
| 114385805 | immunoglobulin light chain variable region [Homo sapiens] | | 127.32 | | 58.33% | | 730 | | 4 | | 1.268 | |
| 323432994 | immunoglobulin variable region [Homo sapiens] | | 217.51 | | 39.71% | | 66 | | 4 | | 1.267 | |
| 323432701 | immunoglobulin variable region [Homo sapiens] | | 234.27 | | 32.12% | | 66 | | 4 | | 1.262 | |
| 58222848 | anti-tetanus toxoid immunoglobulin light chain variable region [Homo sapiens] | | 64.9 | | 22.43% | | 15 | | 2 | | 1.26 | |
| 623409 | Keratin 10 [Homo sapiens] | | 83.17 | | 10.34% | | 8 | | 4 | | 0.769 | |
| 791020 | immunoglobulin light chain variable region [Homo sapiens] | | 113.43 | | 37.96% | | 451 | | 3 | | 0.719 | |
| 179692 | complement component C5, partial [Homo sapiens] | | 335 | | 14.86% | | 9 | | 15 | | 0.713 | |
| 194383506 | unnamed protein product [Homo sapiens] | | 1726.14 | | 70.65% | | 9 | | 43 | | 0.709 | |
| 4176416 | IgG heavy chain [Homo sapiens] | | 226.3 | | 28.17% | | 234 | | 5 | | 0.709 | |
| 221042206 | unnamed protein product [Homo sapiens] | | 745.94 | | 26.91% | | 17 | | 17 | | 0.635 | |
| 380259177 | Chain A, Crystal Structure Of C5b6 | | 326.12 | | 11.77% | | 2 | | 15 | | 0.626 | |
| 223961 | complement C4d | | 272.83 | | 32.65% | | 1 | | 6 | | 0.611 | |
| 553293 | fibronectin, partial [Homo sapiens] | | 40.55 | | 36.73% | | 1 | | 1 | | 0.599 | |
| 235925 | plasma glutathione peroxidase 3 [Homo sapiens] | | 30.74 | | 43.75% | | 5 | | 1 | | 0.509 | |
| 119605337 | hCG36734 [Homo sapiens] | | 48.18 | | 1.61% | | 1 | | 1 | | 0.455 | |
| 530389060 | PREDICTED: PHD finger protein 20-like protein 1 isoform X6 [Homo sapiens] | | 19.84 | | 0.60% | | 1 | | 1 | | 0.453 | |
| 619383 | apolipoprotein D,ApoD [human, plasma, Peptide, 246 aa] | | 165.92 | | 27.24% | | 7 | | 6 | | 0.28 | |
| 33570169 | immunoglobulin kappa chain variable region [Homo sapiens] | | 85.89 | | 33.33% | | 104 | | 2 | | 0.186 | |

Table S5. The DEPs in YDSKpatients (part)

| Accession | Description | Score | Coverage | # Proteins | # Peptides | fold change |  |
| --- | --- | --- | --- | --- | --- | --- | --- |
| 5524087 | lambda 1 immunoglobin light chain variable region [Homo sapiens] | 60.56 | 32.54% | 295 | 3 | 16.73 |  |
| 482673155 | immunoglobulin light chain variable region, partial [Homo sapiens] | 74.44 | 26.17% | 76 | 2 | 3.641 |  |
| 21669541 | immunoglobulin lambda light chain VLJ region [Homo sapiens] | 415.73 | 43.91% | 435 | 10 | 3.628 |  |
| 185364 | immunoglobulin lambda-chain [Homo sapiens] | 433.52 | 48.94% | 509 | 11 | 3.444 |  |
| 21669331 | immunoglobulin kappa light chain VLJ region [Homo sapiens] | 379.06 | 40.37% | 449 | 8 | 2.099 |  |
| 38492827 | Chain B, Globular Head Of The Complement System Protein C1q | 35.27 | 21.21% | 4 | 2 | 2.016 |  |
| 1942682 | Chain B, Deoxy (Beta-(C93a,C112g)) Human Hemoglobin | 276.11 | 69.18% | 145 | 9 | 1.962 |  |
| 125761 | RecName: Full=Ig kappa chain V-I region DEE | 109.81 | 22.22% | 189 | 4 | 1.467 |  |
| 345110894 | Chain A, Complex Of Fcgammariia (Cd32) And The Fc Of Human Igg1 | 467.43 | 74.30% | 4 | 13 | 1.464 |  |
| 7770173 | retinol binding protein 4, PRO2222 [Homo sapiens] | 188.73 | 38.85% | 16 | 5 | 2.754 | |
| 323432682 | immunoglobulin variable region [Homo sapiens] | 165.07 | 26.03% | 438 | 4 | 1.443 |  |
| 13195586 | hemoglobin alpha 1 globin chain [Homo sapiens] | 215.16 | 80.00% | 56 | 6 | 1.435 |  |
| 293651977 | Chain L, Structure Of Human Anti Hiv 21c Fab | 455.05 | 59.91% | 507 | 11 | 1.413 |  |
| 33570169 | immunoglobulin kappa chain variable region [Homo sapiens] | 85.89 | 33.33% | 104 | 2 | 1.393 |  |
| 34526220 | unnamed protein product [Homo sapiens] | 674.86 | 45.76% | 480 | 17 | 1.39 |  |
| 194386718 | unnamed protein product [Homo sapiens] | 151.28 | 31.91% | 1 | 4 | 1.385 |  |
| 323433023 | immunoglobulin variable region [Homo sapiens] | 233.2 | 33.12% | 113 | 5 | 1.369 |  |
| 62113341 | serum albumin [Homo sapiens] | 2351.64 | 71.43% | 13 | 50 | 1.274 |  |
| 332356380 | albumin [Homo sapiens] | 2599.01 | 83.25% | 21 | 55 | 0.798 |  |
| 4837749 | immunoglobulin heavy chain variable region [Homo sapiens] | 31.55 | 10.26% | 1 | 1 | 0.796 |  |
| 197115867 | immunoglobulin heavy chain variable region [Homo sapiens] | 208.07 | 52.29% | 2654 | 7 | 0.795 |  |
| 218512079 | RecName: Full=Ig gamma-2 chain C region | 418.64 | 46.01% | 49 | 12 | 0.795 |  |
| 578809988 | PREDICTED: complement component C6 isoform X4 [Homo sapiens] | 197.99 | 10.81% | 11 | 9 | 0.795 |  |
| 178849 | apolipoprotein E [Homo sapiens] | 500.89 | 41.32% | 18 | 12 | 0.789 |  |
| 194391084 | Kininogen 1[Homo sapiens] | 376.81 | 27.95% | 9 | 2 | 0.712 |  |
| 37811500 | truncated lipopolysaccharide binding protein precursor [Homo sapiens] | 26.87 | 8.48% | 8 | 1 | 0.705 |  |
| 4176416 | IgG heavy chain [Homo sapiens] | 226.3 | 28.17% | 234 | 5 | 0.686 |  |
| 219519923 | Orosomucoid 1 [Homo sapiens] | 185.56 | 29.85% | 6 | 5 | 0.682 |  |
| 323433041 | immunoglobulin variable region [Homo sapiens] | 253.96 | 32.70% | 318 | 7 | 0.68 |  |
| 85567253 | HGF activator, preproprotein [Homo sapiens] | 63.73 | 4.89% | 5 | 2 | 0.669 |  |
| 37694587 | immunoglobulin heavy chain variable region [Homo sapiens] | 44.57 | 16.24% | 1 | 1 | 0.666 |  |
| 90108664 | Chain A, Crystal Structure Of Lipid-Free Human Apolipoprotein A-I | 791.38 | 72.43% | 13 | 21 | 0.662 |  |
| 45015863 | anti-HIV-1 gp120 immunoglobulin X5 light chain [Homo sapiens] | 294.33 | 30.99% | 67 | 5 | 0.655 |  |
| 448262609 | Chain L, Lebrikizumab Fab Bound To Il-13 | 440.63 | 47.71% | 169 | 8 | 0.653 |  |
| 222978 | Ig M Fc | 234.51 | 29.71% | 3 | 6 | 0.647 |  |
| 221042206 | unnamed protein product [Homo sapiens] | 745.94 | 26.91% | 17 | 17 | 0.644 |  |
| 598169 | immunoglobulin heavy chain variable region TTG1K1 Fab [Homo sapiens] | 45.52 | 18.85% | 535 | 2 | 0.638 |  |
| 87299006 | immunoglobulin light chain variable region [Homo sapiens] | 141.25 | 31.13% | 86 | 3 | 0.635 |  |
| 5051025 | immunoglobulin gamma heavy chain variable region [Homo sapiens] | 88.13 | 21.88% | 55 | 2 | 0.628 |  |
| 380259177 | Chain A, Crystal Structure Of C5b6 | 326.12 | 11.77% | 2 | 15 | 0.617 |  |
| 5679478 | immunoglobulin heavy chain variable region [Homo sapiens] | 70.75 | 11.29% | 2996 | 3 | 0.601 |  |
| 791020 | immunoglobulin light chain variable region [Homo sapiens] | 113.43 | 37.96% | 451 | 3 | 0.588 |  |
| 223961 | complement C4d | 272.83 | 32.65% | 1 | 6 | 0.588 |  |
| 218783334 | immunoglobulin light chain [Homo sapiens] | 475.03 | 48.60% | 186 | 8 | 0.574 |  |
| 109693081 | immunoglobulin light chain variable region [Homo sapiens] | 136.37 | 57.94% | 731 | 4 | 0.562 |  |
| 21669509 | immunoglobulin lambda light chain VLJ region [Homo sapiens] | 327.06 | 42.12% | 319 | 10 | 0.561 |  |
| 253723069 | Chain A, Intact Recombined Alpha1-Antitrypsin Mutant Phe 51 To Leu | 1066.78 | 69.29% | 52 | 29 | 0.542 |  |
| 119605337 | hCG36734 [Homo sapiens] | 48.18 | 1.61% | 1 | 1 | 0.327 |  |
| 114385805 | immunoglobulin light chain variable region [Homo sapiens] | 127.32 | 58.33% | 730 | 4 | 0.291 |  |
| 619383 | apolipoprotein D, ApoD [human, plasma, Peptide, 246 aa] | 165.92 | 27.24% | 7 | 6 | 0.169 |  |

Table S6 The DEPs identified with up-regulation in one HIV/AIDS TCM syndrome but with down-regulation in another

| Accession | Description | Score | Coverage | # Proteins | # Peptides | MA/MS | MB/MS |
| --- | --- | --- | --- | --- | --- | --- | --- |
| 6179866 | anti-phospholipid immunoglobulin light chain VL-J-C region [Homo sapiens] | 126.71 | 31.30% | 29 | 4 | 1.226 | 0.793 |
| 87299006 | immunoglobulin light chain variable region [Homo sapiens] | 141.25 | 31.13% | 86 | 3 | 2.02 | 0.635 |
| 63102965 | anti-rabies virus immunoglobulin light chain variable region [Homo sapiens] | 124.27 | 40.19% | 181 | 3 | 1.757 | 0.723 |
| 145942169 | immunoglobulin heavy chain variable region [Homo sapiens] | 90.26 | 35.71% | 2998 | 3 | 1.424 | 0.741 |
| 114385805 | immunoglobulin light chain variable region [Homo sapiens] | 127.32 | 58.33% | 730 | 4 | 1.268 | 0.291 |
| 11118903 | anticardiolipin immunoglobulin light chain, partial [Homo sapiens] | 113.95 | 44.44% | 27 | 3 | 0.8 | 1.224 |
| 4501987 | afamin precursor [Homo sapiens] | 166.26 | 9.18% | 4 | 5 | 0.791 | 1.243 |
| 125761 | RecName: Full=Ig kappa chain V-I region DEE | 109.81 | 22.22% | 189 | 4 | 0.73 | 1.467 |
| 62113341 | serum albumin [Homo sapiens] | 2351.64 | 71.43% | 13 | 50 | 0.722 | 1.274 |
| 93278678 | Chain A, Crystal Structure Of A Cross-Reactive Hiv-1 Neutralizing  Cd4-Binding Site Antibody Fab M18 | 310.75 | 39.91% | 7 | 5 | 0.696 | 2.14 |
| 323433117 | immunoglobulin variable region [Homo sapiens] | 252.79 | 48.78% | 70 | 5 | 0.549 | 1.829 |
| 230651 | Chain A, Structure Of Prealbumin, Secondary, Tertiary and Quaternary Interactions Determined By Fourier Refinement At 1.8 Angstroms | 328.16 | 91.34% | 10 | 8 | 0.484 | 1.387 |
| 33570169 | immunoglobulin kappa chain variable region [Homo sapiens] | 85.89 | 33.33% | 104 | 2 | 0.186 | 1.393 |
